# Use of an innovative model to evaluate mobility in seniors with lower-limb amputations of vascular origin: a pilot study

**DOI:** 10.1186/1471-2318-10-68

**Published:** 2010-09-20

**Authors:** Claude Vincent, Émilie Demers, Hélène Moffet, Hélène Corriveau, Sylvie Nadeau, Catherine Mercier

**Affiliations:** 1 Centre for Interdisciplinary Research in Rehabilitation and Social Integration (CIRRIS), Institut de réadaptation en déficience physique de Québec, 525, Wilfrid-Hamel Blvd East, Québec (Québec), G1M 2S8, Canada; 2 Département de réadaptation, Laval University, Pavillon Ferdinand-Vandry, Quebec City, Quebec, G1K 7P4, Canada; 3 Research Centre on Aging, University Institute of Geriatrics of Sherbrooke, 1036 Belvédère South, Sherbrooke, Quebec J1H 4C4, Canada; 4 Department of Rehabilitation, Faculty of Medicine and Health Sciences, Université de Sherbrooke, 3001, 12th Avenue, Sherbrooke, Quebec, Canada; 5 École de réadaptation, Université de Montréal, c.p. 6128, succursale Centre-ville, Montréal (Québec), H3C 3J7, Canada; 6 Centre de recherche interdisciplinaire de réadaptation, Institut de réadaptation Gingras-Lindsay de Montréal, Canada

## Abstract

**Background:**

The mobility of older individuals has often been only partially assessed, without considering all important aspects such as potential (available) versus effective (used) mobilities and the physical and psychosocial factors that modulate them. This study proposes a new model for evaluating mobility that considers all important aspects, applied here to lower-limb amputees with vascular origin. This model integrates the concepts of potential mobility (e.g. balance, speed of movement), effective mobility (e.g. life habits, movements in living areas) and factors that modulate these two types of mobility (e.g. strength, sensitivity, social support, depression). The main objective was to characterize potential and effective mobility as well as mobility modulators in a small sample of people with lower-limb amputations of vascular origin with different characteristics. The second objective of this pilot study was to assess the feasibility of measuring all variables in the model in a residential context.

**Methods:**

An observational and transversal design was used with a heterogeneous sample of 10 participants with a lower-limb amputation of vascular origin, aged 51 to 83, assessed between eight and 18 months after discharge from an acute care hospital. A questionnaire of participant characteristics and 16 reliable and valid measurements were used.

**Results:**

The results show that the potential mobility indicators do not accurately predict effective mobility, i.e., participants who perform well on traditional measures done in the laboratory or clinic are not always those who perform well in the real world. The model generated 4 different profiles (categories) of participants ranging from reduced to excellent potential mobility and low to excellent effective mobility, and characterized the modulating factors. The evaluations were acceptable in terms of the time taken (three hours) and the overall measurements, with a few exceptions, which were modified to optimize the data collected and the classification of the participants. For the population assessed, the results showed that some of the negative modulators (particularly living alone, no rehabilitation, pain, limited social support, poor muscle strength) played an important role in reducing effective mobility.

**Conclusion:**

The first use of the model revealed interesting data that add to our understanding of important aspects linked to potential and effective mobility as well as modulators. The feasibility of measuring all variables in the model in a residential context was demonstrated. A study with a large number of participants is now warranted to rigorously characterize mobility levels of lower-limb amputees with vascular origin.

## Background

Good mobility, considered very broadly in the field of health (walking, physical activity, participation in society, ability to drive, having access to public transportation, etc.) [1] is the key to autonomy for seniors in their living environment [2]. However, the global profile of mobility in seniors following surgery is not well known, especially in the year following their discharge from an acute care hospital. One of the reasons for this lack of an overall vision is the lack of a model for evaluating mobility that would be comprehensive enough to include all aspects of mobility [3]. Another reason is that certain populations who go through convalescence never undergo rehabilitation. This is particularly the case for people who have had lower-limb amputations of vascular origin in Quebec (Canada), 75% of whom are not referred for rehabilitation [4]. In addition to the absence of clinical follow-up in this population, there has been little research at the international level since most of the mobility studies identified in people with vascular amputation focused on individuals who received prosthetic rehabilitation [5]. What happened to those people who do not receive this specific service? The population concerned is substantial: some 158,000 Americans are hospitalized every year for an amputation [5]. In Canada, the data vary by province. In Quebec (7 million inhabitants), for example, every week more than ten individuals have a lower limb amputated following a vascular problem [4], with the main cause of amputation being diabetes [6-13].

Improved mobility is often related to better quality of life, which is influenced by different factors (movements, energy, pain, sleep, social relationships and emotional reactions) [14]. Amputees suffer from many major health problems, which have a negative effect on their mobility, social participation and quality of life [15]. Therefore it is essential to specify which variables truly influence the mobility of these at-risk groups if we want to take appropriate action in the continuum of services. From this perspective, the purpose of this study was to validate a model to evaluate the mobility of seniors with a lower limb amputated for vascular reasons, and to show that it is possible to take a series of standardized steps in a residential context that cover all three dimensions of the conceptual model.

### Survey of the Literature

A survey of the literature was performed on mobility measures and factors that influence mobility (modulators). Several studies involved monitoring amputees at one year [5,7,11,16], two years [5,16-19], even five years [5,19-21] post-amputation. However, these studies primarily described patients' prognoses, evaluated using mortality rates [1,5-8,11,16,17,20-25] and the level of using assistive devices or a prosthetic leg [7,17,19,23,25,26]. Other studies focused on capabilities [27-32] (e.g. walking distance, walking speed, muscle strength, balance), participation in activities of daily life (ADL) at home [24,33], participation in community activities and the ability to drive a car [24,25]. However, most of these studies focused strictly on patients who had been fitted with a prosthetic or only on a few aspects related to mobility. No study documented mobility in the broadest sense (e.g. level of physical activity, time spent on daily activities), inside and outside the home of patients with amputations of vascular origin.

A review of the literature conducted by Rommers et al [3] on this patient group identified 18 studies measuring the ability of individuals (e.g. walking without assistance, with a cane, with a walker, using a wheelchair) and 17 studies measuring activities performed in a real context (e.g. walking outside the home, climbing stairs, walking in the neighbourhood, performing a number of household activities, driving a car). These authors noted that some of these studies had simultaneously used measurements of capabilities and activities as indicators of mobility, but were not exhaustive, i.e., did not cover all capabilities, activities and contextual factors. Surprisingly, in 2008, i.e., 11 years later, the same observations can be made: no study can be found on the longitudinal monitoring of mobility of vascular amputees that documents both capabilities and the performance of activities in the home and the community.

Twenty-four modulating factors that interfere with the underlying capabilities of mobility and the performance of activities overall were identified in the literature survey. Among these factors, certain studies highlighted the risks of co-morbidity found in vascular amputees: high blood pressure, heart disease, stroke, obesity and chronic obstructive pulmonary disease [1,5,8,15,17,22,24]. Smoking, drinking and inactivity are also some of the risk factors that negatively impact the precarious physical health of these individuals [8-11,17,21]. Moreover, the risk of amputation increases after age 60, and seems to be higher in men than women [6,8,22]. A longitudinal study also showed that a lack of social support, measured one month post-amputation, predicts symptoms of depression at one year and two years post-amputation [34]. This last study demonstrated the importance of using biopsychosocial intervention models to facilitate long-term adjustment, i.e., psychologically, socially and physically, especially for phantom pain [34].

Other studies identified several factors that positively influenced the resumption of certain significant activities for vascular amputees, such as social support [8,18,26-30], satisfaction with the prosthesis [23,28,31] or assistive devices [32,33,35], proactive coping strategies [23,26,28-30,36] and an optimistic personality [28,37]. It has also been acknowledged that the level of amputation below the knee, absence of pain, and possibility of maintaining social relationships support the underlying capabilities of mobility and performance of activities [23]. Lastly, environmental barriers (accessibility, attitude of other people, availability of resources, social support) interfere with the capabilities and activities of people with arm or leg amputations [38,39].

Given all the modulating factors identified, a model for evaluating mobility from a broader perspective was proposed by a team of researchers from various disciplines with a view to documenting the evolution of this population.

### Model for Evaluating Mobility

**Figure 1:**
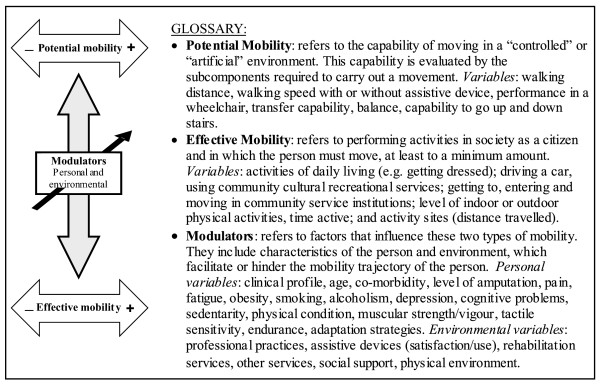
**Model for evaluating mobility**.

Our team developed a model to evaluate mobility that takes into account two types of mobility that may change differently over time depending on different modulating factors experienced by people with a lower-limb amputation of vascular origin. The definitions and concepts are illustrated in Figure 1. Our team consists of 15 researchers from various disciplines, all of whom are interested in different aspects of mobility (see Table 1). They attended ten brainstorming meetings [40] over a period of a year-and-a-half, carried out in prepared face-to-face meetings.

**Table 1:** Profile of the Researcher Team (n = 15)

Characteristics	Number of Researchers
Sex	
Male	6
Female	9
Expertise with clientele	
Geriatric	5
Adult	10
Research	
in the laboratory	7
in an actual environment	8
Type of basic training	
Physiotherapy	6
Occupational therapy	4
Physical activity or Exercise science physiology	2
Social worker	1
Engineering	1
Psychology	1
Doctoral education	
Rehabilitation	8
Neurobiology	2
Public health	1
Social work	1
Exercise science and Exercise physiology and biology	1
Geriatrics (clinical sciences) or gerontology	2
Applied human sciences	1
Interest in assistive devices	
walking aids (cane, walker, tripod stick, quadripod stick, crutches)	9
Wheelchair and motorised mobility aids	2
GPS and actimetry system	1
prosthesis	3
Affiliated with	
University de Montreal	4
University of Sherbrooke	4
Laval University (Quebec City) McGill University (Montreal)	6 1
Number of years as a researcher (Post-Ph.D)	
1-5 years	4
6-10 years	5
11 years and +	6

In this model, effective mobility means the actual participation of people in society, in a "real environment", and is a determining factor in their quality of life. This mobility is modulated by various factors related to health (e.g. active rehabilitation, community support), the person's condition (e.g. comorbidity) and the physical and human environment (e.g. social support, use of assistive devices). Potential mobility, which is mainly based on certain physical capabilities required to carry out a movement, indicates the potential of a person to move in a "laboratory or clinical environment". Interaction between the variables of these two types of mobility and modulators can vary greatly from individual to individual. In this study, it was hypothesized that as multiple modulators influence mobility, potential mobility would be a poor predictor of effective mobility. For example, a participant in a power wheelchair may have had both legs amputated, may be unable to to walk, and may have no balance or low upper trunk mobility (low potential mobility), but may perform home ADL by substitution or with assistive technology, may participate in community activities and may be able to visit friends with adaptation of the human and physical environments (high effective mobility). A better understanding of the model variables and their interactions will help identify meaningful and useful data to better direct individuals and their families to the most appropriate services for them.

### Study objectives

The main objective was to characterize potential and effective mobility indicators as well as mobility modulators in a small sample of people with lower-limb amputations of vascular origin with different characteristics. The second objective of this pilot study was to verify the feasibility of measuring all variables in the model in a residential context.

## Methods

An observational design with multiple measures taken at a single point in time was used as part of this feasibility study. Since the objectives of this pilot study were to test the model and assess feasibility, all outcome measures including questionnaires, physical tests, ADL and community activity outcomes were assessed once between between eight and 18 months following discharge from an acute care hospital. A delay of at least eight months between amputation and assessment was chosen to make sure that participants had time to adjust to their amputation and resume their activities, in order to get a representative picture of their effective mobility. The research protocol was approved by the Research Ethics Committee of the participating institution (Hôpital St-François D'Assise) and all participants provided their informed consent.

### Recruitment of Participants

A heterogeneous sample (n = 10) of various ages, including ambulatory and non-ambulatory participants, using different assistive devices to move, was selected for the study (mean age: 71 years, 8 men and 2 women). There were three recruitment phases based on a non-probabilistic sampling method. Of the 60 files selected by the archivist in an urban acute care hospital, 35 were selected based on the following eligibility criteria: be 55 years of age or older, have had an amputation of vascular origin of a lower limb above the ankle at least eight months following discharge from an acute care hospital and live within a 75-km radius of the recruitment site. People with severe cognitive or oral communication problems were excluded. Of these 35 files, only 22 were retained following a careful review of the medical files by a research assistant. All of them were contacted by phone to verify their eligibility. It was learned that some had died by the time of the study, while others no longer met the selection criteria. Finally, the eligibility of 18 people was confirmed and ten of them agreed to participate (acceptance rate of 55%).

### Data Collection Procedure

Each participant was met at his/her home, and underwent an evaluation (three hours maximum) by a research assistant and a physiotherapist. All participants first filled in a consent form and then did the questionnaires and physical tests based on a standardized procedure. To minimize the effects of fatigue, tests involving intellectual and physical effort were alternated. The level of fatigue was evaluated using a visual analogue scale (VAS) for every four tests to ensure that participants were able to continue the evaluation. The left extremity of the VAS indicates no fatigue (0) and the right one, lots of fatigue (5 inches long). If the result exceeded 2.5 inches, a longer pause would be required. The tests or questions with a strong emotional dimension were left until the end.

### Measuring Instruments

Ten instruments associated with the ***modulators ***of mobility were used to document the influence of 24 variables identified as factors influencing mobility [1,8-11,17,23,34,36,38,39,41-47]. Note that the term "negative" modulator refers to the idea that a personal or environmental factor, when it reaches a certain threshold, may limit or hinder mobility.

The *sociodemographic, clinical, physical and psychosocial characteristics *of subjects were collected using a questionnaire developed for the study. Negative modulators included age over 60 [25], an amputation below the knee [19], fatigue felt when performing ADL [19], daily consumption of cigarettes and alcohol [48,49], living alone or in a care centre [50], a physical environment that is not very accessible [38,39], dissatisfaction with the technical aids used [47], presence of another physical problem and lack of services (rehabilitation, etc.)[39].

The *Charlson Comorbidity Scale *adapted to a geriatric clientele [51] was used to identify medical conditions via the medical record. A score of 2 or more was considered to be a negative modulator [51].

The *Interpersonal Support Evaluation List *(ISEL), developed by Cohen et al. [52] and adapted by McColl and Skinner [53], measuring three types of support--instrumental (7 items), informational (6 items) and emotional (9 items) was used. It includes a four-level Likert scale, from 0 to 3, as well as a satisfaction scale for each type of support. The total score (average of three types of support) is the variable used. A score lower than 1 indicating low support is considered to be a modulator-influencing variable.

The *MOS Social Support Survey *[54] was used as a social support measurement. The French version of this tool has good psychometric qualities. It was validated in a rehabilitative context of patients with cardiovascular disease [55]. It includes 19 questions covering five types of support: tangible, emotional, affective, positive social interaction and informational. Scales of 0 to 5 ("never" to "always") showed an average score of 5 for each type of support. A score of < 75/95 was considered to be a negative modulator [56].

The *Ways of Coping Questionnaire *(WCQ) [53] was used to measure the adaptability of the individual following difficult events, such as returning home following discharge from an acute care hospital. Its internal coherence is good. The abridged version with 21 items is divided into three different aspects, i.e.: 1) distancing and avoidance, 2) looking for social support, and 3) positive re-evaluation and problem solving. A Likert-type scale allows to measure the level of use of different adaptive strategies. Adaptation was considered to be a negative modulator when Aspect 1 received an average score of > 1.5/3 or when aspects 2 and 3 received average scores of < 1.5 for aspects 2 or 3 [53].

The *Modified Brief Pain Inventory *(BPI) [57,58] was used to evaluate pain intensity and how this pain interferes with the person's activity. It was used with various types of patients including those with neuropathic pain [59-61]. A French version of the BPI was validated [62-66]. Scores of > 5/10 (Question 5) or > 7/10 (Question 9a) on the Likert scale were considered to be negative modulators [67].

A *Body Mass Index *(BMI) of more than 30 was considered to be a negative modulator [68,69]. Height (cm) and weight (kg) were taken from in the patient file.

The *Yesavage Geriatric Depression Scale *was used [70,71]. Depression was identified starting with a score of 11/30 downward, with 92-95% sensitivity and 84-89% specificity. Above the threshold (11/30), it was considered to be a negative modulator [72].

The Jamar *dynamometer *[73] is valid and reliable [74] for measuring hand-grip strength, which is also considered to be an overall strength index for the individual. Based on the standards established according to age, strength (the average of two tests) in the 30th percentile or less was a negative modulator [74].

The *Semmes-Weinstein Monofilament Test *(10 g) was used to test protection sensitivity of the intact foot [75]. Four sites of application were evaluated (big toe, top of the first, third and fifth metacarpals), at a rate of two actual stimulations plus one factice per site. The rest-retest reliability is good [75-77]. As soon as a monofilament was not felt, regardless of the site of application, sensitivity was considered to be a negative modulator [78].

Four instruments were used to evaluate ***potential mobility***, i.e., capabilities to perform various activities requiring movement [27-31,42,79-85].

The *Locomotor Capabilities Index (LCI) *[86] is a measurement of the perception of a person's capability to perform activities with and without his/her prosthesis. The patient is asked whether he can do seven basic activities (from 'getting up from a chair' to 'going downstairs'), and seven advanced activities (from 'picking up an item from the ground when he is standing using a prosthesis' to 'walking while carrying an item'). Each task is rated on a scale (0 = no, 1 = yes with help, 2 = yes with surveillance, 3 = yes). Two sub-scores out of 21 were produced and a total score of 42. This potential mobility indicator "failed" if the score was equal to or less than 21/42, given that this threshold indicates frequent difficulties and the need for technical or human assistance [87].

The *Timed Up and Go Test *[88] (TUG) measures the time required to get up from a chair, walk three metres and then sit down again. The inter-rater and test-retest reliabilities are very high among ambulatory lower-limb amputees [30]. For individuals in wheelchairs, one task of the Wheelchair Skills Test [89,90] was used instead of the TUG, i.e., the transfer from a wheelchair to a flat surface (e.g. bed, chair), moving forward three metres in the wheelchair, turning, returning and once again going from the wheelchair to the to the initial surface. This indicator of potential mobility was considered to be "failed" if TUG > 14 seconds in both versions (prostheses and with assistive devices for walking) [88]. For the wheelchair-adapted TUG, since no standard cut-point has been established or recommended in the literature, the threshold was set at > 34 seconds, based on the average (n = 6) obtained during the pilot project.

The *Berg Balance Test *(BBT) [91] is a measurement of seated and standing balance. It is made up of 14 subtests, rated from 0 to 4. A score lower than 45 is predictive of the risk of multiple falls.

The *Amputee Mobility Predictor *(AMP) [92] is a measurement that predicts the movement capabilities of lower-limb amputees. Its psychometric qualities were demonstrated in English and French [93]. The AMP evaluates 21 situations requiring seated or standing balance, during movement or when reaching for objects. Scales from 0 to 2 are used, except for the last item. A failing mark in this potential mobility indicator was when the score was under 25/47. Under this threshold, the subject is unstable, loses balance and cannot reach objects [92].

Three instruments associated with ***effective mobility ***cover the evaluation of activities in a real environment [3,33,94] and the level of physical activity able to be performed.

The *Assessment of Life Habits *(LIFE-H) [95,96], abridged version 3.1, was used as the main way of measuring outcomes of effective mobility. This tool was developed from the Handicap Production Process [97] and evaluates handicap situations that hinder social participation. It includes 200 items that could be grouped daily activities as well as social roles. The score varies from 0 (high level of handicap situations) to 9 (optimal level of social participation) for each item. The reliabilities were excellent for the overall score studied in a group of 84 seniors who had lost autonomy (CCI and confidence intervals at 95%: 0.95 (0.91 to 0.98) (test-retest) and 0.89 (0.80 to 0.93) (inter-rater) [98]. This effective mobility indicator was considered to have "failed" for daily activities and for social roles if their average score is < 7/9, given that, at this threshold, the person can only carry out a given activity with an assistive device, or layout modifications or requires human assistance [96].

The *Life Space Assessment (LSA) *[99] identifies the regular mobility patterns during the month preceding the evaluation. Five levels of movement are assessed, from inside the home to outside the city. Its score varies from 0 (complete restriction of one's mobile space) to 120 (no restriction of one's mobile space). Validity, reliability and sensitivity to change in the LSA were studied in ambulatory seniors and wheelchair users [99,100]. Metric properties in the French-Canadian version are good [101]. This effective mobility indicator was deemed to have "failed" if the mobility space scores were below the standard values for people aged 65 and more [78].

The *Human Activity Profile *(HAP) [102] measures the capability to perform different levels of physical activity. It is a questionnaire with 94 questions, is self-administered and validated for different clienteles [102-104]. It begins with questions on the activities that require low energy expenditure and ends with questions associated with high energy levels. Each item must be answered using one of the following three statements: I still do this activity, I have stop doing this activity and I have never done this activity. The maximum activity score (MAS), which corresponds to the last item that the subject had done was selected. This potential mobility indicator was considered low if the MAS was lower than the standard values for the age [105].

### Data Analysis

Descriptive statistics were calculated for all variables using SPSS software (version 15.0 for Windows Grad Pack) for the group of ten participants. The results were then grouped into three distinct themes: potential mobility, effective mobility and modulators for each participant (see Additional file 1). Given the limits or standards specified in the literature to judge whether a test has been failed, the scores deemed "negative ☹" and "positive" were transformed into dichotomous data (0: fail/negative, 1: success/positive). All items (0 or 1) were then added to quantify the potential mobility (number of successful items out of a maximum of four possible items assessed: LCI, TUG a or b, TUG c, AMP), effective mobility (number of successful items out of a maximum of four possible items assessed: LIFE-F personal care, LIFE-F social roles, LSA-F, HAP) and modulators (number of successful items out of a maximum of 24 possible items assessed). Then, all the total scores on these three indicators were weighted out of four (4), when they were not based on four items. This weighting of scores on the same scale allowed us to compare the two types of mobility and modulators, and also compare the patients. For example, if one patient did not do the TUG-c, his/her total potential mobility indicator based on 3 items (instead of four) would be weighted out of four, to make it possible to compare across the indicators and the other patients. If one patient only anwered 20 of the 24 possible modulator items, his/her total modulator score would also be weighted out of four. The scores weighted by category were interpreted as follows: low/negative (< 1), moderate (1 to < 3) and high/positive (3 to 4). These outcomes were then graphed to show the profile of each participant.

## Results

The sociodemographic, clinical, physical and psychosocial characteristics are shown in Additional file 1 as modulator variables. Data analysis revealed that six participants showed low potential mobility (weighted score 0/4), three were moderate (1.33/4 or 2.66/4) and one revealed high potential mobility (weighted score: 4/4). Modulators were moderate for nine participants (lower score 7/16 and higher score 17/24, weighted scores 1.75/4, 1.83/4) and positive for a single participant (subject #8; weighted score: 3.17/4). For effective mobility, three participants highlighted in grey in Additional file 1 showed high effective mobility (weighted score: 4/4 for 2 subjects; 3/4 for one subject), six were moderate (weighted score: 1/4 for three subjects; 2/4 for three subjects) while only one had low effective mobility (weighted score: 0/4).

Additional file 1 reveals that some of the negative modulators (particularly living alone, no prosthetic rehabilitation, pain, low social support, poor muscle strength) played an important role in "low effective mobility". Those negative modulators were not present for the three participants with high effective mobility.

Additional file 1 presents the individual scores on the various tests and weighted scores for the three categories of measurements. Subject #1 had missing data for three assessments (not evaluated/n.e.) because the research assistant had to stop after three hours (ethical committee's requirement). Also, there are missing data (-) for two assessments because the research team decided to replace them for the last five subjects by more adapted tests; this is the case for the ISEL (replaced by the MOS) and the Berg Balance Test (replaced by the AMP). Finally, there are missing data (not applicable/n.a.) for subjects who did not use a wheelchair or prosthesis; this is the case for the TUG, AMP and LCI.

**Figure 2:**
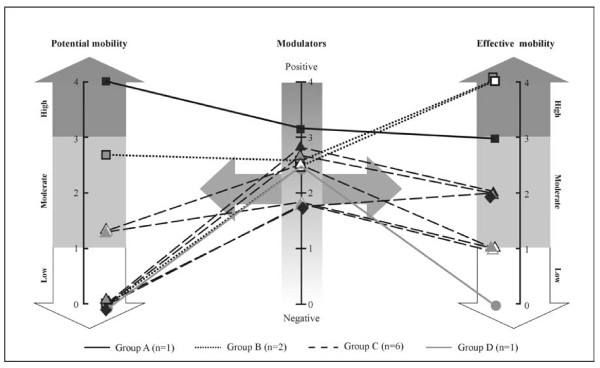
**Graphic representation of the scores of ten participants in the study in terms of potential and effective mobility and modulators**. Participant categories: Group A (n = 1): **high effective mobility**, positive modulators and high potential mobility. Group B (n = 2): **high effective mobility**, moderate modulators and low/moderate potential mobility. Group C (n = 6): **moderate effective mobility**, moderate modulators and low/moderate potential mobility. Group D (n = 1): **low effective mobility**, moderate modulators and low potential mobility.

Figure 2 provides a graphic representation of the weighted scores for each of the three categories of measurements for the ten subjects. By grouping the subjects based on their weighted effective mobility score, four distinct groups were formed. First, two groups attained high effective mobility despite the fact that their potential mobility and their number of positive modulators are different: Group A (n = 1) presents high potential mobility as well as high positive modulators, whereas Group B (n = 2) displays potential mobility ranging from low to moderate, and moderate modulators. Next, Group C (n = 6) presented moderate effective mobility with low potential mobility and moderate modulators. Lastly, Group D (n = 1) demonstrated low effective mobility with low potential mobility and moderate modulators.

## Discussion

This pilot study collected preliminary data on potential and effective mobility indicators as well as the modulators for these mobilities in people with a lower-limb amputation of vascular origin. It also assessed the feasibility of measuring, in a residential context, all variables in the proposed model for evaluating mobility. The data collected led to dividing the participants into four subgroups with different profiles of effective and potential mobility. Since we only had 10 subjects, we do not believe the number of participants under each profile is a valid representation of the number expected. However, it certainly indicates the substantial heterogeneity in mobility of the lower-limb vascular amputee population. It seems that our model was able to detect different patterns of level of mobility (described by others as capabilities [27-32]), participation in ADL at home [24,33] and community activities [24,25].

The seven participants who showed low/moderate effective mobility are different than the ones with high effective mobility, when considering the type of modulators reported but not the number of modulators. In fact, we observed that it was only in groups C and D (n = 7) showing moderate and low effective mobility that three or more of a specific subset of modulators were observed: living alone (n = 3), no prosthetic rehabilitation (n = 3), low social support (n = 2), no coping strategy to obtain social support (n = 4); general pain (n = 3), little strength in one of their arms (n = 5) and low sensitivity in the remaining limb (n = 3). As well, in group C and D, seven participants reported a low level of activity intensity. In previous studies, often focusing on one aspect, these modulators were also identified as risk factors for poor mobility [8,18,34,36,37,67,74,78,105]. Our results suggest that a given subject could present a complex situation where negative modulators interact with potential mobility in such a way that low effective mobility is observed. Clearly, this justifies taking a global approach using multidimensional assessments to better understand the complexity of factors underlying mobility in a larger group of amputees.

These initial outcomes of the different mobility groups suggest many possible areas of research. In particular, it appears important to assess the impact of resources and services, which are potential mobility modulators, on the profile of effective and potential mobility of amputees following their stay in an acute care hospital. In order to better document the temporal trajectory of mobility in vascular amputees, a large-scale longitudinal study is needed. These data would be useful for making recommendations on the main mobility modulators and could guide decision-making for resources and services to make available to this population. A better knowledge of mobility modulators and their impact at different times of motor recovery would make it possible to identify areas to develop physical and psychosocial activities adapted to the needs of these individuals. Knowledge regarding the different levels and types of mobility in vascular amputees is fragmented compared to other populations that could present similar needs for care and services (e.g. people with post-stroke hemiparesis). A vision of the mobility profile for all vascular amputees is essential for implementing strategies to facilitate a high level of effective mobility in these people. Data from representative samples would be helpful for practitioners and community partners in order to determine: 1) which patients (profile) are most likely to benefit from intervention; 2) when is the best time to intervene in the first nine months following the amputation; and 3) what is the optimal target for intervention (e.g. potential mobility or a subset of modulators) to improve effective mobility. This study offers a conceptual framework to establish such a profile for mobility and modulators of this mobility.

This study also helped confirm the feasibility of the proposed protocol. First, it was possible to collect most of the data by studying participants in their homes. All tests and questionnaires were administered without too much participant fatigue and within the anticipated period (maximum of three hours, minimum of 2 hours 45 minutes). All participants scored < 2.5/5 on the fatigue visual analogue scale, all the time, and none required a longer break between the tests. This study also resulted in certain improvements, which are currently being implemented. Two tests were replaced after meeting with half the participants. The *Interpersonal Support Evaluation List *[52] was too difficult to administer, and was therefore replaced by the *MOS Social Support Survey *[54]. The *Berg Balance Test *[83] was replaced by the *Amputee Mobility Predictor *[92] in order to be able to better document balance when sitting in a wheelchair. Further details on *Ways of Coping *were also broughtby the researchers to facilitate its administration, such as asking more directly "how returning home was a potentially stressful event". A rating (0 to 5) on overall satisfaction with all assistive devices used for movement was also added. In addition, future studies should involve recording the subjects' weight at home, instead of taking it from the file, in order to have the most current weight of the subject. It will then be possible to use anthropomorphic tables to add the weight of the missing part to the subject's weight. This is necessary in order to use the BMI standard tables developed for the entire body. For "potential mobility", we should note that a measurement instrument had to be developed (wheelchair version of the TUG). Although the results are promising, further information on the procedure for this modified version of the TUG is necessary. For example, it will have to be specified on which side to make the transfer (ipsilateral or contralateral to the amputation), and which parts of the wheelchair to take into account when crossing the finish line. The possibility of breaking down the final score (total time) into the time spent on certain activities must be considered, including transfer time and movement time. The ICL questionnaire was also adapted to people without prostheses in order to take into account mobility using other assistive devices. Investigations will be required to validate and establish standards for the adaptations of the known instruments.

## Conclusions

The proposed model for evaluating mobility with its three dimensions (potential and effective mobility and their modulators) seems promising for characterizing mobility of lower-limb amputees. Preliminary data revealed that discrepancies might exist between potential mobility and effective mobility for a given individual, supporting the need to assess both types of mobility and the modulators. This pilot study provided the basis for a large-scale longitudinal assessment of mobility aiming to characterize profiles of mobility in vascular amputees over time. Ideally, in a longitudinal context, data should be collected sooner after the amputation and at different times to determine the evolution of the mobility indicators. Then, it would be possible to propose recommendations regarding their resource and service needs to optimize their mobility and quality of social participation.

## Competing interests

The authors declare that they have no competing interests.

## Authors' contributions

The rehabilitation RQRV group 2006-2009 participated in the conception of the design of the study, data analysis and in all of the research meetings. CV was one of the two principal investigators, assumed the coordination of all the research meetings, conceived the design of the study, submitted the research protocol for its financing, participated in the data analysis, outlined and drafted the manuscript with a summer student in research (ED). ED managed the database, performed the data analysis and drafted more specifically the result section of the manuscript and commented the manuscript. HM was one of the two principal investigators, assume the coordination of the research assistants, conceived design the study, participated in the data analysis and commented the manuscript. HC, SN and CM also commented the manuscript. These authors contributed equally to this work. All authors approved the final manuscript.

## Pre-publication history

The pre-publication history for this paper can be accessed here:

http://www.biomedcentral.com/1471-2318/10/68/prepub

## Supplementary Material

Additional file 1 Results of the Variables for Modulators, Potential Mobility and Effective Mobility Click here for file

## References

[B1] Institut de Recherche en Santé du Canada [Internet] Institut du Vieillisement. Initiative mobilité et vieillissement. Résultats des consultations en ce qui concerne les priorités de recherche et les activités favorisant l'application des connaissances issues de la recherche 2007 http://www.irsc-cihr.gc.ca/f/33610.html [cited 2008 Aug 27]

[B2] Collin C Collin J Mobility after lower limb amputation Br J Surg 1996 83 1 132 8 10.1002/bjs.1800830152 7648138

[B3] Rommers GM Vos LDW Groothoff JW Eisma WH Mobility of people with lower limb amputations: scales and questionnaires: a review Clin Rehabil 2001 15 92 102 10.1191/026921501677990187 11237166

[B4] Ministère de la santé et des services sociaux du Québec [Internet] Info-Med-Echo Bulletins d'information sur l'hospitalisation de soins courte durée au Québec 2006 http://www.msss.gouv.qc.ca [cited 2006 Sep 15]

[B5] Dillingham TR Pezzin LE Mackenzie EJ Limb amputation and limb deficiency in the United States: an epidemiological analysis South Med J 2002 95 875 83 12190225 10.1097/00007611-200208000-00018

[B6] Armona GA Hoffmeyer P Herrmann FR Herrmann FR Vaucher J Tschopp O Lacraz A Vischer UM Major lower limb amputations in the elderly observed over ten years: the role of diabetes and peripheral arterial disease Diabetes Metab 2005 31 5 449 54 10.1016/S1262-3636(07)70215-X 16357788

[B7] Eskelinen E Lepäntalo M Hietala EM Sell H Kauppila L Mäenpää I Pitkänen J Salminen-Peltola P Leutola S Eskelinen A Kivioja A Tukiainen E Lukinmaa A Brasken P Railo M Lower limb amputations in Southern Finland in 2000 and trends up to 2001 Eur J Vasc Endovasc Surg 2004 27 2 193 200 10.1016/j.ejvs.2003.10.011 14718903

[B8] Rieden RA The geriatric amputee Physical Medicine and Rehabilitation Clinics of North America 2005 16 1 179 95 10.1016/j.pmr.2004.06.004 15561550

[B9] Dillingham TR Pezzin LE Mackenzie EJ Discharge destination after dysvascular lower-limb amputations Arch Phys Med Rehabil 2003 84 11 1662 68 10.1053/S0003-9993(03)00291-0 14639567

[B10] Vaccaro O Lodato S Mariniello P De Feo E Diabetes-related lower extremity amputations in the community: a study based on hospital discharge diagnoses Nutr Metab Cardiovasc Dis 2002 12 6 331 6 12669680

[B11] Dillingham TR Pezzin LE Shore AD Reamputation, mortality, and health care costs among persons with dysvascular lower-limb amputations Arch Phys Med Rehabil 2005 86 3 480 6 10.1016/j.apmr.2004.06.072 15759232

[B12] Canadian Diabetes Association [Internet] Prévention et coûts du diabète. Document 02-125 #124007 2002 http://www.diabetes.ca/Files/Francais/prevalence_et_couts_du_diabete.pdf [cited 2007 Nov 12]

[B13] Alami LEL Lazghad A Chadli A Ghomari HEL Farouqi AEL Marouan F Waziz A Zryouil B Les facteurs pronostiques dans l'amputation du pied chez le diabétique Med Chir Pied 2005 21 4 134 8 10.1007/s10243-005-0058-8

[B14] Pell JP Donnan PT Fowkes FG Ruckley CV Quality of life following lower limb amputation for peripheral arterial disease Eur J Vasc Surg 1993 7 4 448 51 10.1016/S0950-821X(05)80265-8 8359304

[B15] Health Canada [Internet] Le diabète au Canada. Centre de prévention et de contrôle des maladies chroniques. Direction générale de la santé de la population et de la santé publique 2002 http://www.phac-aspc.gc.ca/publicat/dic-dac2/francais/05contents_f.html [cited 2007 Nov 12]

[B16] Lacroix P Aboyans V Medeau L Preux PM Bertin F Cornu E Laskar M Survie à long terme des amputés vasculaires âgés Arch Mal Coeur Vaiss 2000 93 10 1189 93 11107477

[B17] Buzato MA Tribulatto EC Costa SZ Zorn WG van Bellen B Major amputations of the lower leg. The patients two years later Acta Chir Belg 2002 102 4 248 52 12244903 10.1080/00015458.2002.11679306

[B18] Williams RM Ehde DM Smith DG Czerniecki JM Hoffman AJ Robinson LR A two-year longitudinal study of social support following amputation Disabil Rehabil 2004 26 14-15 862 74 10.1080/09638280410001708878 15497915

[B19] Pardasaney PK Sullivan PE Portney LG Mankin HJ Advantage of limb salvage over amputation for proximal lower extremity tumors Clin Orthop Relat Res 2006 444 201 8 10.1097/01.blo.0000195413.16150.bc 16449916

[B20] Aulivola B Hile CN Hamdan AD Sheahan MG Veraldi JR Skillman JJ Campbell DR Scovell SD LoGerfo FW Pomposelli FB Jr Major lower extremity amputation: outcome of a modern series Arch Surg 2004 139 4 395 9 10.1001/archsurg.139.4.395 15078707

[B21] Mayfield JA Reiber GE Maynard C Czerniecki JM Caps MT Sangeorzan BJ Survival following lower-limb amputation in a veteran population J Rehabil Res Dev 2001 38 3 341 5 11440266

[B22] Cutson TM Bongiorni DR Rehabilitation of the older lower limb amputee: a brief review J Am Geriatr Soc 1996 44 11 1388 93 8909359 10.1111/j.1532-5415.1996.tb01415.x

[B23] Fusetti C Senechaud C Merlini M Quality of life of vascular disease patients following amputation Ann Chir 2001 126 5 434 9 10.1016/S0003-3944(01)00541-7 11447794

[B24] Stewart CP Jain AS Cause of death of lower limb amputees Prosthet Orthot Int 1992 16 2 129 32 1408672 10.3109/03093649209164325

[B25] Taylor SM Kalbaugh CA Blackhurst DW Hamontree SE Cull DL Messich HS Robertson RT Langan EM York JW Carsten CG Snyder BA Jackson MR Youkey JR Preoperative clinical factors predict postoperative functional outcomes after major lower limb amputation: an analysis of 553 consecutive patients J Vasc Surg 2005 42 2 227 35 10.1016/j.jvs.2005.04.015 16102618

[B26] Van de Weg FB van der Windt DA A questionnaire survey of the effect of different interface types on patient satisfaction and perceived problems among trans-tibial amputees Prosthet Orthot Int 2005 29 3 231 9 10.1080/03093640500199679 16466153

[B27] Brooks D Parsons J Hunter JP Devlin M Walker J The 2-minute walk test as a measure of functional improvement in persons with lower limb amputation Arch Phys Med Rehabil 2001 82 10 1478 83 10.1053/apmr.2001.25153 11588757

[B28] Hatfield AG Beyond the 10-m time: a pilot study of timed walks in lower limb amputees Clin Rehabil 2002 16 2 210 4 10.1191/0269215502cr484oa 11911519

[B29] Franchignoni F Brunelli S Orlandini D Ferriero G Traballesi M Is the Rivermead Mobility Index a suitable outcome measure in lower limb amputees?-A psychometric validation study J Rehabil Med 2003 35 3 141 4 10.1080/16501970310010493 12809197

[B30] Schoppen T Boonstra A Groothoff JW de Vries J Goeken LN Eisma WH The Timed 'up and go' test: reliability and validity in persons with unilateral lower limb amputation Arch Phys Med Rehabil 1999 80 7 825 8 10.1016/S0003-9993(99)90234-4 10414769

[B31] Munin MC Espejo-De Guzman MC Boninger ML Fitzgerald SG Penrod LE Singh J Predictive factors for successful early prosthetic ambulation among lower-limb amputees J Rehabil Res Dev 2001 38 4 379 84 11563490

[B32] Collin C Wade DT Cochrane GM Functionnal outcome of lower limb amputees with peripheral vascular disease Clin Rehabil 1992 6 1 13 21

[B33] Treweek SP Condie ME Three measures of functional outcome for lower limb amputees: a retrospective review Prosthet Orthot Int 1998 22 3 178 85 9881605 10.3109/03093649809164482

[B34] Hanley MA Jensen MP Ehde DM Hoffman AJ Patterson DR Robinson LR Psychosocial predictors of long-term adjustment to lower-limb amputation and phantom limb pain Disabil Rehabil 2004 26 882 93 10.1080/09638280410001708896 15497917

[B35] Demers L Wessels RD Weiss-Lambrou R Ska B De Witte LP An international content validation of the Quebec User Evaluation of Satisfaction with Assistive Technology (QUEST) Occup Ther Int 1999 6 3 159 75 10.1002/oti.95

[B36] Desmond DM MacLachlan M Coping strategies as predictors of psychosocial adaptation in a sample of elderly veterans with acquired lower limb amputations Soc Sci Med 2006 62 1 208 16 10.1016/j.socscimed.2005.05.011 15990211

[B37] Gallagher P MacLachlan M Positive meaning in amputation and thoughts about the amputated limb Prosthet Orthot Int 2000 24 3 196 204 10.1080/03093640008726548 11195354

[B38] Ephraim PL MacKenzie EJ Wegener ST Dillingham TR Pezzin LE Environmental barriers experienced by amputees: the Craig Hospital Inventory of Environmental Factors-Short Form Arch Phys Med Rehabil 2006 87 3 328 33 10.1016/j.apmr.2005.11.010 16500165

[B39] Pasquina PF Bryant PR Huang ME Roberts TL Nelson VS Flood KM Use and satisfaction with prosthetic limb devices and related services Arch Phys Med Rehabil 2004 85 723 29 10.1016/j.apmr.2003.06.002 15129395

[B40] Fontana A Frey JH Denzin NK, Lincoln YS The interview: From structured questions to negociated text The handbook of qualitative research 2000 2 Thousands Oaks CA, Sage 645 72

[B41] Ploeg AJ Lardenoye JW Vrancken Peeters MP Breslau PJ Contemporary series of morbidity and mortality after lower limb amputation Eur J Vasc Endovasc Surg 2005 29 633 7 10.1016/j.ejvs.2005.02.014 15878543

[B42] Gauthier-Gagnon C Grise MC Tools for outcome measurement in lower limb amputee rehabilitation Montreal (Qc): Centre de documentation, Institut de réadaptation de Montréal, Montréal, Qc, Canada

[B43] Bosmans JC Suurmeijer TP Hulsink M van der Schans CP Geertzen JHB Dijkstra PU Amputation, phantom pain and subjective well-being: a qualitative study Int J Rehabil Res 2007 30 1 8 10.1097/MRR.0b013e328012c953 17293714

[B44] Desmond DM Coping, affective distress, and psychosocial adjustment among people with traumatic upper limb amputations J Psychosom Res 2007 62 15 21 10.1016/j.jpsychores.2006.07.027 17188116

[B45] Horgan O MacLachlan M Psychosocial adjustment to lower-limb amputation: a review Disabil Rehabil 2004 26 837 50 10.1080/09638280410001708869 15497913

[B46] Desmond DM MacLachlan M Affective distress and amputation-related pain among older men with long-term, traumatic limb amputations J Pain Symptom Manage 2006 31 362 8 10.1016/j.jpainsymman.2005.08.014 16632084

[B47] Boone DA Coleman KL Use of the Prosthesis Evaluation Questionnaire (PEQ) J Prosthet Orthot 2006 18 68 79 10.1097/00008526-200601001-00008

[B48] Levy LA Smoking and peripheral vascular disease. Podiatric medical update Clin Podiatr Med Surg 1992 9 1 165 71 1735060

[B49] Gamba MA Gotlieb SL Bergamaschi DP Vianna LA Lower extremity amputations in diabetic patients: a case-control study Rev saúde pública 2004 38 3 399 404 10.1590/S0034-89102004000300010 15243670

[B50] Mahoney JE Eisner J Havighurst T Gray S Palta M Problems of older adults living alone after hospitalization J Gen Intern Med 2000 15 9 611 9 10.1046/j.1525-1497.2000.06139.x 11029674 PMC1495595

[B51] Charlson M Pompei P Ales ML MacKenzie CR A new method of classifying comorbidity in longitudinal studies: development and validation J Chronic Dis 1987 40 5 373 93 10.1016/0021-9681(87)90171-8 3558716

[B52] Cohen S Mermelstein R Kamarck T Hoberman HM Sarason IG, Sarason BR Measuring the functional components of social support Social Support: Theory, Research and Applications 1985 Dordrecht, Martinus Nijhoff 73 94

[B53] McColl M Skinner H Measuring psychological outcomes following rehabilitation Can J Public Health 1992 83 2 12 8 1468043

[B54] Anderson D Bilodeau B Deshaies G Gilbert M Validation canadienne-français du MOS Social Support Survey Can J Cardiol 2005 21 10 867 73 16107910

[B55] Sherbourne CD Steward AL The MOS Social Support Survey Soc Sci Med 1991 32 6 705 14 10.1016/0277-9536(91)90150-B 2035047

[B56] Moskovitz DN Maunder RG Cohen Z McLeod RS MacRae H Coping behavior and social support contribute independently to quality of life after surgery for inflammatory bowel disease Dis Colon Rectum 2000 43 4 517 21 10.1007/BF02237197 10789749

[B57] Cleeland CS Ryan KM Pain assessment: global use of the Brief Pain Inventory Ann Acad Med Singapore 1994 23 129 38 8080219

[B58] Poundja J Fikretoglu D Guay S Brunet A Validation of the French version of the brief pain inventory in Canadian veterans suffering from traumatic stress J Pain Symptom Manage 2007 33 6 720 6 10.1016/j.jpainsymman.2006.09.031 17531912

[B59] Tan G Jensen MP Thornby JI Shanti BF Validation of the Brief Pain Inventory for chronic nonmalignant pain J Pain 2004 5 133 7 10.1016/j.jpain.2003.12.005 15042521

[B60] Smith BH Torrance N Bennett MI Lee AJ Health and quality of life associated with chronic pain of predominantly neuropathic origin in the community Clin J Pain 2007 23 143 9 10.1097/01.ajp.0000210956.31997.89 17237663

[B61] McDermott AM Toelle TR Rowbotham DJ Schaefer CP Dukes EM The burden of neuropathic pain: results from a cross-sectional survey Eur J Pain 2006 10 127 35 10.1016/j.ejpain.2005.01.014 16310716

[B62] Poundja J Fikretoglu D Guay S Brunet A Validation of the French version of the brief pain inventory in Canadian veterans suffering from traumatic stress J Pain Symptom Manage 2007 33 720 6 10.1016/j.jpainsymman.2006.09.031 17531912

[B63] Tyler EJ Jensen MP Engel JM Schwartz L The reliability and validity of pain interference measures in persons with cerebral palsy Arch Phys Med Rehabil 2002 83 236 9 10.1053/apmr.2002.27466 11833028

[B64] Marshall HM Jensen MP Ehde DM Campbell KM Pain site and impairment in individuals with amputation pain Arch Phys Med Rehabil 2002 83 1116 9 10.1053/apmr.2002.33121 12161833

[B65] Raichle KA Osborne TL Jensen MP Cardenas D The reliability and validity of pain interference measures in persons with spinal cord injuries J Pain 2006 7 179 86 10.1016/j.jpain.2005.10.007 16516823

[B66] Tyler EJ Jensen MP Engel JM Schwartz L The reliability and validity of pain interference measures in persons with cerebral palsy Arch Phys Med Rehabil 2002 83 236 9 10.1053/apmr.2002.27466 11833028

[B67] Jensen MP Smith DG Ehde DM Robinsin LR Pain site and the effects of amputation pain: further clarification of the meaning of mild, moderate, and severe pain Pain 2001 91 3 317 22 10.1016/S0304-3959(00)00459-0 11275389

[B68] Santé Canada [Internet] Lignes directrices canadiennes pour la classification du poids chez les adultes. Ministre des Travaux publics et Services gouvernementaux du Canada 2003 http://www.hc-sc.gc.ca/fn-an/nutrition/weights-poids/guide-ld-adult/weight_book_tc-livres_des_poids_tm_f.html [cited 2007 Nov 12].

[B69] Pinzur M Freeland R Juknelis D The association between body mass index and foot disorders in diabetic patients Foot Ankle Int 2005 26 5 375 7 15913521 10.1177/107110070502600506

[B70] Yesavage JA Geriatric Depression Scale Psychopharmacol Bull 1988 24 4 709 11 3249773

[B71] U.S Agency for Health Care Policy and Reasearch Post-Stroke rehabilitation : Assessment, referral, and patient management - Quick Reference Guide for Clinicians Agency for Health Care Policy and Research/AHCPR Publication No. 95-0663 1995 7 12 407 623 7627210

[B72] Bourque P Blanchard L Vézina J: Étude psychométrique de l'Échelle de dépression gériatrique Can J Aging 1990 9 348 55

[B73] Desrosiers J Hébert R Bravo G Dutil E Comparison of the Jamar dynamometer and the Martin vigorimeter for grip strength measurements in a healthy elderly population Scand J Rehabil Med 1995 27 3 137 43 8602475

[B74] Mathiowetz V Weber K Volland G Kashman NJ Reliability and validity of grip and pinch strength evaluations J Hand Surg [Am] 1984 9 2 222 6 6715829 10.1016/s0363-5023(84)80146-x

[B75] Semmes J Weinstein S Ghent L Teuber HL Somatosensory changes after penetrating brain wounds in man 1960 Cambridge MA, Harvard University Press

[B76] Bell-Krotoski J Tomancik E The repeatability of testing with Semmes-Weinstein monofilaments J Hand Surg [Am] 1987 12 155 61 3805636 10.1016/s0363-5023(87)80189-2

[B77] Bell-Krotoski JA Buford WL The Force/Time Relationship of Clinically Used Sensory Testing Instruments J Hand Ther 1988 10 4 297 309 10.1016/s0894-1130(97)80045-2 9399179

[B78] Peel C Sawyer Baker P Roth DL Brown CJ Brodner EV Allman RM Assessing mobility in older adults: the UAB Study of Aging Life-Space Assessment Phys Ther 2005 85 10 1008 119 16180950

[B79] Loiret I Paysant J Martinet N André JM Evaluation of amputees Ann Readapt Med Phys 2005 48 6 307 16 15932782 10.1016/j.annrmp.2005.03.009

[B80] Ryall NH Eyres SB Neumann VC Bhakta BB Tennant A Is the Rivermead Mobility Index appropriate to measure mobility in lower limb amputees? Disabil Rehabil 2003 25 143 53 10.1080/0963828021000024951 12648004

[B81] Russek AS Management of lower extremity amputees Arch Phys Med Rehabil 1961 42 687 703 14495389

[B82] Collin C Collin J Mobility after lower limb amputation Br J Surg 1995 82 1010 1 10.1002/bjs.1800820803 7648138

[B83] Datta D Ariyaratnam R Hilton S Timed walking test - an all-embracing outcome measure for lower limb amputees? Clin Rehabil 1996 10 3 227 32 10.1177/026921559601000307

[B84] Berg KO Wood-Dauphinee SL Williams JI Maki B Measuring balance in the elderly: validation of an instrument Can J Public Health 1992 83 2 7 11 1468055

[B85] Hanspal RS Fisher K Assessment of cognitive and psychomotor function and rehabilitation of elderly people with prostheses British Med J 1991 302 940 10.1136/bmj.302.6782.940 PMC1669445 2032036

[B86] Grisé MC Gauthier-Gagnon C Martineau GG Prosthetic profile of people with lower extremity amputation: conception and design of a follow-up questionnaire Arch Phys Med Rehabil 1993 74 8 862 70 10.1016/0003-9993(93)90014-2 8347072

[B87] Gauthier-Gagnon C Grisé MC Prosthetic profile of the amputee questionnaire: validity and reliability Arch Phys Med Rehabil 1994 75 1309 14 7993169

[B88] Podsiadlo D Richardson S The Timed "Up and Go": A test of basic functional mobility for frail elderly persons J Am Geriatr Soc 1991 39 2 142 8 1991946 10.1111/j.1532-5415.1991.tb01616.x

[B89] Routhier F Kirby RL Demers L Vincent C Guérette C Dessureault D Raymond D Westwood D Measurement properties of the French-Canadian version of the Wheelchair Skills Test: Preliminary results Canadian Seating and Mobility Conference, Oct 4-6 2006 Toronto, Ontario 106 http://www.csmc.ca/docs/archives/2006_archive/2006CSMC_Proceedings_Final.pdf

[B90] Routhier F Demers L Kirby RL Pervieux I Depa M De Serres L Loiselle F Dessureault D Inter-rater and Test-Retest Reliability of the French-Canadian Wheelchair Skills Test (Version 3.2): Preliminary Findings Proceedings from a conference. RESNA 2007 - 30th International Conference on Technology & Disability: Research, Design and Practice 2007 Phoenix, AZ: Siegler S & Smith RO

[B91] Berg K Maki B Williams JI Holliday PJ Wood-Dauphinee S Clinical and laboratory measures of postural balance in an elderly population Arch Phys Med Rehabil 1992 73 11 1073 80 1444775

[B92] Gailey RS Roach KE Applegate EB Cho B Cunniffe B Licht S Maguire M Nash MS The amputee mobility predictor: an instrument to assess determinants of the lower-limb amputee's ability to ambulate Arch Phys Med Rehabil 2002 83 5 613 27 10.1053/ampr.2002.32309 11994800

[B93] Tremblay A Allen MF Enfin nous avons la possibilité d'évaluer le potentiel d'appareillage d'une personne amputée! Journée scientifique et professionnelle de l'Institut de réadaptation en déficience physique de Québec, 2007 June 1 2007 Québec (Qc), Canada 10 http://www.irdpq.qc.ca/Evenements/joursc2008/pdf/interieur_finalaveccalendrier_prog2007.pdf

[B94] Boulias C Meikle B Pauley T Devlin M Return to driving after lower-extremity amputation Arch Phys Med Rehabil 2006 87 1183 8 10.1016/j.apmr.2006.06.001 16935052

[B95] Desrosiers J Noreau L Robichaud L Fougeyrollas P Rochette A Viscogliosi C Validity of the Assessment of Life Habits (LIFE-H) in older adults J Rehabil Med 2004 36 177 82 10.1080/16501970410027485 15370734

[B96] Fougeyrollas P Noreau L La Mesure des habitudes de vie: version abrégée (MHAVIE 3.1) 2002 Lac St-Charles QC, Réseau international sur le Processus de production du handicap (RIPPH) 22

[B97] Fougeyrollas P Les modèles explicatifs des conséquences des maladies: Le processus de production de handicap Réseau international de la CIDIH 1992 6 2 14 28

[B98] Noreau L Desrosiers J Robichaud L Fougeyrollas P Rochette A Viscogliosi C Measuring social participation: Reliability of the LIFE-H among older adults with disabilities Disabil Rehabil 2004 26 346 52 10.1080/09638280410001658649 15204486

[B99] Baker PS Bodner EV Allman RM Measuring life-space mobility in community-dwelling older adults J Am Geriatr Soc 2003 51 11 1610 4 10.1046/j.1532-5415.2003.51512.x 14687391

[B100] Meyers AR Anderson JJ Miller DR Shipp K Hoenig H Barriers, facilitators, and access for wheelchair users: substantive and methodologic lessons from a pilot study of environmental effects Soc Sci Med 2002 55 1435 46 10.1016/S0277-9536(01)00269-6 12231020

[B101] Auger C Demers L Gélinas I Routhier F DeRuyter F Development of a French-Canadian version of Life-Space Assessment (LSA-F): content validity, reliability and applicability for power mobility device users Disabil Rehabil Assist Technol 2009 4 1 31 41 10.1080/17483100802543064 19172479

[B102] Fix A Daughton D Human activity profile: professional manual 1988 Odessa FL, Psychological Assessment Resources, Inc

[B103] Daughton DM Fix AJ Kass I Bell CW Patil KD Maximum oxygen consumption and the ADAPT quality-of-life scale Arch Phys Med Rehabil 1982 63 620 2 7149948

[B104] Davidson M de Morton N A systematic review of the Human Activity Profile Clin Rehabil 2007 21 151 62 10.1177/0269215506069475 17264109

[B105] Bilek LD Venema DM Willett GM Lyden ER Use of the Human Activity Profile for estimating fitness in persons with arthritis Arthritis Rheum 2008 59 5 659 64 10.1002/art.23572 18438897

