# Supplementary material for: Use of an innovative model to evaluate mobility in seniors with lower-limb amputations of vascular origin: a pilot study
Source: BMC Geriatr. 2010 Sep 20;10:68. doi: 10.1186/1471-2318-10-68 (PMC2955596; doi:10.1186/1471-2318-10-68)
Supplement: Additional file 1 — Results of the Variables for Modulators, Potential Mobility and Effective Mobility [file 1471-2318-10-68-S1.PDF]

Table 2 Results of the Variables for Modulators, Potential Mobility and Effective Mobility

**MODULATORS**						↓	↓		↓		
1. Sociodemographic, clinical, physical and psychosocial characteristics	Score/ Patient	#1	#2	#3	#4	#5	#6	#7	#8	#9	#10
❑ Age[5,7,10]		55	51	☹74	☹73	☹81	☹75	☹73	☹83	☹64	☹82
❑ Sex[5,7,10]		☹H	☹H	F	☹H	☹H	☹H	☹H	☹H	☹H	F
❑ Level of amputation[16]		☹ Tr-fem	☹ Tr-fem	☹ Tr- fem	☹ Tr-fem	☹ Tr-fem.	Tr-tib.	☹ Tr-fem.	Tr-tib	☹ Tr-fem	☹ Tr-fem
❑ Other physical disability		No	No	No	No	No	No	No	No	No	☹ Yes
❑ BMI[63]		Normal	☹Class 2	☹Class 3	Normal	☹Class 3	☹Class 3	☹109kg	70kg	☹130kg	☹66kg
❑ Fatigue during ADL[116]		No	No	Depends	No	No	Depends	No	No	Depends	Depends
❑ Tobacco[117,118]		☹ Oui	Non	☹ Oui	Non	Non	Non	Non	Non	☹ Oui	Non
❑ Alcohol[117,118]		Never	Occas.	Occas.	Weekly	Occas.	Occas.	Never	Weekly	Occas.	Never
❑ Living environment after amputation[119]		☹Alone at home	Room- mate	☹Alone at home	Spouse partner	Spouse/ partner	Spouse/ partner	☹Alone at home	Spouse partner	Spouse partner	☹Dom. seul
❑ Prosthetic rehabilitation[109]		☹ Half	Yes	☹No	Yes	Yes	Yes	☹No	Yes	☹No	☹No
❑ Adapted physical environment [36,109]		☹No	Yes	Yes	Yes	Yes	Yes	Yes	Yes	Yes	Yes
❑ Overall satisfaction with assistive devices used (S or I; /5) [35]		W;WC;P I ☹	C;W;P S	WC;SC I ☹	P;C;W; CR;WC S	P;C;CR; WC S.	P;WC S.	W,WC 5/5	P,C,WC 4/5	WC,SC 4/5	WC, mWC 4/5
2. Charlson Co-morbidity Scale[50]		☹4	☹6	☹6	☹3	☹4	☹2	1	☹2	☹ 3	☹2
3. Interpersonal Support Eval. List[103]	/3	2.11	2.73	☹1.77	2.8	3	-	-	-	-	-
3a. MOS[51]	/95	-	-	-.	-	-	95	78	94	☹45	95
4. Coping (adaptative) strategies[53]*	/3										
Avoidance (/3) (☹ >1.5/3)		n.e.	0	0.33	0	☹1.67	1.33	0	0.17	0.83	0.17
Social support (/3) (☹ <1.5/3)		n.e.	☹0	☹0.67	☹0.83	☹0.67	☹0.17	1.83	☹0.67	☹0.67	☹1.33
Positive (/3) (☹ <1.5/3)		n.e.	☹0	1.89	☹0.67	1.67	☹1.89	2.33	☹1.44	☹0.11	☹1.25
5. Modified Brief Pain Inventory [54]											
a) General pain (Question 5)	/10	n.e.	☹5	☹5.5	1	0	n.e.	☹5	0	1	0
b) Pain interference (Question 9)	/10	n.e.	0	3.17	0	0	n.e.	0	0	0	0
6. Yesavage Geriatric Depression Scale[64]	/30	n.e.	4	9	1	10	n.e.	8	0	3	4
7. Jamar Dynamometer[72]	(kg)										
Left arm		☹25	☹25	☹18	27.7	30	31	☹13.3	25.7	45.3	☹6
Right arm		☹27	☹ 6.3	☹21	31.7	34	28.67	25.6	27.7	46	☹4
8. Semmes-Weinstein Monofilaments[74]	/24										
Number of tests passed	/8	n.e.	8	n/a	☹1	☹5	☹6	☹7	8	8	☹7
Number of shams passed	/4	n.e.	4	n/a	4	☹3	☹3	☹2	4	4	☹3

**TOTAL MODULATORS passed/done**		6/16	16/24	9/22	17/24	15/24	13/21	14/24	19/24	14/24	10/24
Modulator weighting, /4		***1.5/4***	***2.67/4***	***1.64/4***	***2.83/4***	***2.5/4***	***2.48/4***	***2.33/4***	***3.17/4***	***2.33/4***	***1.67/4***

POTENTIAL MOBILITY

Questionnaires and Tests	Score/ Patient	#1	#2	#3	#4	#5 ↓	#6 ↓	#7	#8 ↓	#9	#10
9. Locomotor Capability Index[27]											
a) prothesis version	/42	n/a	☹16	n/a	☹13	☹18	☹14	n/a	37	n/a	n/a
b) version without prothesis	/42	☹15	n/a	☹4	n/a	n/a	n/a	☹2	n/a	☹ 1	☹ 2
10. Timed Up and Go[67]											
a) ambulatory version	(s)	☹22.13	☹27.48	n/a	☹31.64	☹31.12	9.25	n/a	11.01	n/a	n/a
b) version adapted for wheelchair	(s)	n/a	☹56.32	☹ refuse	16.1	n/a	-	☹44	23.12	32.3	33.93
c) Change in assistive device	(s)	n/a	n/a	n/a	☹29.4	n/a	n/a	n/a	n/a	n/a	n/a
11. Berg Balance Test[104]	/56	☹20	☹35	☹	☹35	☹41	-	-	-	-	-
11a. AMP[70]											
a) score without prothesis	/43	-	-	-	-	-	n/a	☹ 9	n/a	☹12	☹10
b) score with prothesis	/47	-	-	-	-	-	41	n/a	39	n/a	n/a
**TOTAL POTENTIAL MOBILITY, passed/done**		0/3	0/4	0/3	0/4	0/3	2/3	0/3	4/4	1/3	1/3
Potentialmobility weighting/4		***0/4***	***0/4***	***0/4***	***0/4***	***0/4***	***2.66/4***	***0/4***	***4/4***	***1.33/4***	***1.33/4***

EFFECTIVE MOBILITY

Questionnaires	Score/ Patient	#1	#2	#3	#4	#5 ↓	#6 ↓	#7	#8 ↓	#9	#10
12. Assessment of Life Habits (LIFE-H)[48,77]											
Personal care (six indicators)	/9	7.13	7.97	☹6.80	7.60	7.96	7.53	☹6.08	7.47	☹6.25	☹6.83
Social roles (six indicators)	/9	☹3.70	☹4.77	☹4.27	☹6.54	7.11	8.09	☹3.77	☹6.74	☹4.28	☹4.67
13. Life Space Assessment (LSA-F)[79]	/120	☹14	☹29	☹31	☹29	51	58.5	☹17.5	48	48	49
14. Human Activity Profile (HAP)[81]	/94	71	72	53	61	73	75	☹36	73	☹36	☹36
**TOTAL EFFECTIVE MOBILITY. passed/done on/4**		***2/4***	***2/4***	***1/4***	***2/4***	***4/4***	***4/4***	***0/4***	***3/4***	***1/4***	***1/4***
Fatigue-visual analogue scales (average of four measurements)	/5	0.12	0	0.07	1.13	0	0	0.09	0.09	0.27	0

☹ = negative risk factor, low score or below the norm; n.e: not evaluated, because the evaluation meeting did not exceed three hours. n/a. the test was not applicable for the participant, e.g. no healthy leg. - : it was not stipulated in the administration protocol at this time.
Tr-tib = transtibial, Tr-fem = transfemoral, Occas = occasional W = walker; WC = wheelchair; mWC = motorized wheelchair; C = single cane; CR = crutches; P = prothesis; SC = scooter. Columns with ↓ indicate participants with high EFFECTIVE mobility indicator.
